# Intersectionality of Disability Status, Family Income, Race, and Ethnicity with Taking a Leave of Absence During Medical School

**DOI:** 10.1007/s11606-025-09662-9

**Published:** 2025-07-25

**Authors:** Mytien Nguyen, Suchita Rastogi, Karina Pereira-Lima, Amy Addams, Christopher J. Moreland, Dowin B. Boatright, Lisa M. Meeks

**Affiliations:** 1 Department of Immunobiology, Yale School of Medicine, 333 Cedar Street, New Haven, CT 06510 USA; 2 Stanford School of Medicine, Palo Alto, CA USA; 3 Department of Anesthesiology, University of Michigan Medical School, Ann Arbor, MI USA; 4 Association of American Medical Colleges, Washington, DC USA; 5 Department of Internal Medicine, Dell Medical School at the University of Texas at Austin, Austin, TX USA; 6 Department of Emergency Medicine, New York University Grossman School of Medicine, New York, NY USA; 7 Department of Family Medicine, University of Michigan Medical School, Ann Arbor, MI USA; 8 Department of Medical Education, University of Illinois College of Medicine, Chicago, IL USA

## INTRODUCTION

Interruptions during medical training can hinder a trainee’s path to becoming a physician.^1^ Low-income students, racially underrepresented in medicine (URiM) students, and medical students with disabilities (MSWD) are disproportionately more likely to take a leave of absence (LOA),^2,3^ a factor associated with higher attrition and reduced residency match.^1,2^ Despite these disparities, little is known about the cumulative impact of these intersecting identities on LOA rates. Here, we examined the prevalence of LOA in a national sample of U.S. medical graduates by disability status, income, race, ethnicity, and their intersections.

## METHODS

Deidentified data was obtained from the Association of American Medical Colleges Graduation Questionnaire (GQ) respondents in academic years 2020 to 2022. Students self-reported sex, disability status, race, ethnicity, and family income. Race and ethnicity were re-categorized into 8 groups: White, Asian, Black, Hispanic, American Indian/Alaska Native/Native Hawaiian/Pacific Islander (AIAN/NHPI), Other, and Multiracial. Family income categorizes were re-categorized into quintiles, as previously reported:^4^ ≥ $200,000, $125,000-$199,999, $100,000-$124,999, $75,000-$99,999, $50,000-$74,999, and < $50,000. Low-income was defined as students with family income ≤ $74,999.

Our study cohort included graduates who took either the 1991 or 2015 version of the Medical College Admission Test (MCAT). To minimize bias, students’ first-attempt raw score quartiles were computed separately for each version, and a unified quartile variable was created by aligning the quartiles across test versions.

Multivariate logistic regression was used to estimate the odds of LOA by disability status, race, ethnicity, family income, and their intersections, adjusting for MCAT quartiles and sex. The study was deemed exempt by the University of Michigan Institutional Review Board. Analyses were performed using Stata 18.0 (StataCorp) and followed the STROBE reporting guidelines.

## RESULTS

Among 50,139 graduates who completed the 2020–2022 GQ, 35,982 (71.7%) had complete sociodemographic data and were included in the study cohort. Compared to nondisabled students, a higher proportion of MSWD took LOA (13.7% vs. 4.2%, aOR: 3.42, 95%CI: 3.04–3.84, Table 1). Students with family income < $50,000 were 40% more likely to take LOA compared to students with family income ≥ $200,000 (aOR: 1.40, 95%CI: 1.19–1.65). Compared to White students, Asian (aOR: 1.84, 95%CI:1.63–2.07), Black (aOR: 1.23, 95%CI:1.02–1.47), and Hispanic (aOR: 1.46, 95%CI:1.26–1.69) students were more likely to take LOA (Table 1).

**Table 1:** Leave of Absence Among 2020 to 2022 MD-granting Medical School Graduates

	**Total**	**Leave of absence**	**Adjusted odds ratio (95% CI)**
*Total*	*N* = *35,982*	*1,782 (4.9%)*	
Disability status			
No	33,006 (91.7%)	1,491 (4.2%)	*Ref*
Yes	2,976 (8.3%)	436 (13.7%)	3.42 (3.04–3.84)
Childhood income			
≥ $200,000	6,630 (18.4%)	259 (3.9%)	*Ref*
$125,000-$199,999	6,216 (17.3%)	223 (3.5%)	0.90 (0.75–1.09)
$100,000-$124,999	4,865 (13.5%)	203 (4.1%)	1.02 (0.84–1.23)
$75,000-$99,999	5,992 (16.7%)	267 (4.4%)	1.03 (0.86–1.23)
$50,000-$74,999	5,417 (15.1%)	298 (5.5%)	1.17 (0.98–1.39)
< $50,000	6,862 (19.1%)	532 (7.7%)	1.40 (1.19–1.65)
Race and ethnicity			
White	19,708 (54.8%)	809 (3.8%)	*Ref*
Asian	7,833 (21.8%)	501 (6.0%)	1.84 (1.63–2.07)
Black	2,208 (6.1%)	164 (7.2%)	1.23 (1.02–1.47)
Hispanic	3,688 (10.2%)	299 (7.8%)	1.46 (1.26–1.69)
AIAN/NHPI	74 (0.2%)	8 (10.1%)	2.03 (0.96–4.32)
Multiracial	1,356 (3.8%)	74 (5.0%)	1.23 (0.96–1.58)
Unknown/Other	1,115 (3.1%)	72 (6.0%)	1.37 (1.06–1.76)
Sex			
Male	16,713 (46.4%)	823 (4.6%)	*Ref*
Female	19,269 (53.6%)	1,104 (5.3%)	1.06 (0.96–1.16)
MCAT quartile			
1^st^ (lowest)	10,448 (29.0%)	925 (8.4%)	*Ref*
2	9,590 (26.7%)	400 (3.9%)	0.48 (0.42–0.54)
3	8,325 (23.1%)	366 (4.1%)	0.50 (0.44–0.58)
4^th^ (highest)	7,619 (21.2%)	236 (2.8%)	0.35 (0.30–0.42)

**Figure 1:**
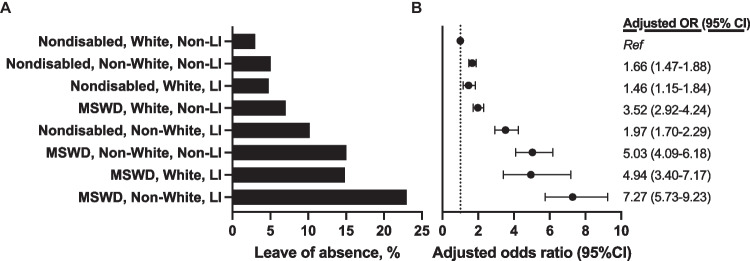
Leave of absence by disability status, race, ethnicity, and childhood income. **A** Rates of leave of absence during medical school by intersectional disability status, race and ethnicity, and childhood income. **B** Adjusted odds of leave of absence by intersectional groups, adjusting for sex and MCAT quartiles. Abbreviations: MSWD: medical students with disability; LI: low-income.

LOA rates increased with increasing intersecting marginalized identities. Compared to nondisabled White non-low-income students, non-White low-income MSWD were 7-times more likely to take LOA (22.9% vs. 3.0%, aOR: 7.27, 95%CI 5.73–9.23, Fig. 1). There was no significant interaction between disability status, family income, and race (*p* = 0.65).

## DISCUSSION

In this study, MSWD were 3-times more likely to take LOA than nondisabled students, with compounded risk among students with intersecting low-income and non-White identities. Notably, one in five non-White low-income MSWDs took a LOA.

LOA decisions are often driven by challenges related to insufficient accommodations, burnout, mental health challenges, and financial hardship—factors that disproportionately impact disabled, non-White, and low-income students.^5,6^ The downstream consequences of LOA—including financial loss, disruption in housing and insurance, social isolation from peers, stigma,^7^ reduced access to school-based resources, and lower residency match rates^1^—highlight the urgent need to address factors contributing to high LOA rates among in non-White low-income MSWD.

While a LOA can be an essential step toward recovery and academic success—offering a crucial opportunity to prioritize wellbeing and manage academic or personal challenges—the process is frequently complicated by lack of clarity, limited logistical and financial support, and institutional stigma.^7^

To mitigate harm and support student wellbeing during an LOA, institutions must develop comprehensive LOA policies that include bridge funding, continued access to mental health care and reintegration programs that include individualized support from faculty and staff.^7^ Reframing LOAs as a standard and supported part of medical education journey is also critical to reducing stigma.

Findings from this study add to the growing body of literature supporting intersectionality theory, which suggests that multiple social and institutional barriers can intersect to intensify the overall burden—or allostatic load—for MSWD. Limitations include reliance on self-reported data and the inability to explore causal relationships. Future research should explore the structural and institutional pathways that contribute to elevated LOA risk and examine the long-term impacts of LOA on trainee outcomes and workforce retention.
